# PREDICTION OF THE NUMBER OF AND CARE COSTS FOR DISABLED OLDER ADULTS FROM 2020 TO 2050 IN URBAN AND RURAL AREAS IN CHINA

**DOI:** 10.1093/geroni/igz038.3117

**Published:** 2019-11-08

**Authors:** liangwen zhang, Ya Fang

**Affiliations:** 1 Center for Aging Health Research, School of Public Health, Xiamen University, Xiamen, China, China; 2 School of Public Health, Xiamen University, Xiamen, China

## Abstract

Disability for the elderly has become a crucial policy concerns in rapidly aging Asia counties, especially in China. This study aimed to predict the trend of the number of and care costs for disabled older adults from 2020 to 2050 in urban and rural areas in China. Population Administration Decision Information System was used to predict the population of China by urban and rural areas and age group from 2020 to 2050. Monte Carlo simulation and Policy Simulation Model were used to estimate the number and care costs of disabled elderly between urban and rural areas, based on the Chinese latest census data, statistical yearbook, and national survey database. The total disabled population rises rapidly from 43.75 million in 2020 to 91.4 million in 2050, of which 69.7% were urban adults. Compared with the values in 2020, the growth rates of the adults with mild, moderate and severe disabilities were 108%, 104% and 120% in 2050, respectively. The value were 167% and 39% in urban and rural areas, respectively. By 2050, the total care costs increase from 538.0 billion yuan in 2020 to 8530.8 billion yuan, of which 80.2% occurs in urban areas. The predicted results indicate that the numbers and care costs for disabled older adults increase sharply from 2020 to 2050, especially in urban areas of China. It provided a series of evidence for the establishment of the long-term care insurance system in China.

